# Impact of glucocorticoids and rapamycin on autophagy in Candida glabrata-infected macrophages from BALB/c mice

**DOI:** 10.3389/fimmu.2024.1367048

**Published:** 2024-03-15

**Authors:** Zhenghui Yang, Xinyi Wang, Tianxiang Dong, Wei-Jia Zhao, Hongbin Li

**Affiliations:** Department of Dermatology and Venereology, First Affiliated Hospital of Kunming Medical University, Kunming, Yunnan, China

**Keywords:** Candida glabrata, macrophage, autophagy, glucocorticoids, LC3-associated phagocytosis

## Abstract

**Objective:**

In the defense against microorganisms like Candida albicans, macrophages recruit LC3(Microtubule-associated protein 1A/1B-light chain 3) to the periplasm, engaging in the elimination process through the formation of a single-membrane phagosome known as LC3-associated phagocytosis (LAP). Building on this, we propose the hypothesis that glucocorticoids may hinder macrophage phagocytosis of Candida glabrata by suppressing LAP, and rapamycin could potentially reverse this inhibitory effect.

**Methods:**

RAW264.7 cells were employed for investigating the immune response to Candida glabrata infection. Various reagents, including dexamethasone, rapamycin, and specific antibodies, were utilized in experimental setups. Assays, such as fluorescence microscopy, flow cytometry, ELISA (Enzyme-Linked Immunosorbent Assay), Western blot, and confocal microscopy, were conducted to assess phagocytosis, cytokine levels, protein expression, viability, and autophagy dynamics.

**Results:**

Glucocorticoids significantly inhibited macrophage autophagy, impairing the cells’ ability to combat Candida glabrata. Conversely, rapamycin exhibited a dual role, initially inhibiting and subsequently promoting phagocytosis of Candida glabrata by macrophages. Glucocorticoids hinder macrophage autophagy in Candida glabrata infection by suppressing the MTOR pathway(mammalian target of rapamycin pathway), while the activation of MTOR pathway by Candida glabrata diminishes over time.

**Conclusion:**

Our study elucidates the intricate interplay between glucocorticoids, rapamycin, and macrophage autophagy during Candida glabrata infection. Understanding the implications of these interactions not only sheds light on the host immune response dynamics but also unveils potential therapeutic avenues for managing fungal infections.

## Introduction

Long-term use of glucocorticoids significantly increases the risk of invasive candidiasis (1). Despite this known association, the impact of glucocorticoids on macrophage-mediated Candida phagocytosis remains unclear. In our study, we discovered that glucocorticoids inhibit macrophage secretion of inflammatory cytokines and impede autophagy. Notably, glucocorticoid treatment initially inhibits the MTOR pathway in macrophages, followed by time-dependent attenuation and eventual activation. Furthermore, alternative pathways beyond MTOR may be involved in glucocorticoid-mediated impairment of macrophage autophagy.

The impaired macrophage phagocytosis of Candida glabrata under glucocorticoid influence was evident. Interestingly, the autophagy agonist rapamycin progressively enhanced macrophage autophagy against Candida glabrata in the presence of glucocorticoids. This leads us to hypothesize that glucocorticoids hinder macrophage phagocytosis by suppressing autophagy, introducing a potential novel mechanism underlying glucocorticoid-mediated immunosuppression and the development of invasive candidiasis.

Invasive candidiasis, predominantly involving Candida albicans, presents a global health concern with a mortality rate of 50-71% (2). Long-term glucocorticoid use is a prevalent risk factor, affecting 41.24% of invasive candidiasis patients (1). Despite being crucial for various treatments, glucocorticoids exhibit associations beyond fungal infections, exacerbating conditions like fungal keratitis and contributing to Pneumocystis carinii pneumonia (3, 4).

Macrophages, essential innate immune cells, play a pivotal role in recognizing and responding to various pathogens (5). Autophagy, a dynamic cellular process, is crucial for immune system maintenance (6). Our investigation focuses on the impact of glucocorticoids on macrophage phagocytosis of Candida glabrata, revealing a temporal inhibition of the MTOR pathway and subsequent hindrance of autophagy, ultimately attenuating macrophage phagocytosis. This sheds light on a potential mechanism underlying glucocorticoid-mediated immunosuppression against invasive candidiasis.

Cryptococcus neoformans, a fungus capable of establishing a latent infection within macrophages, highlights the intricate interplay between macrophages and fungal pathogens (7). Recognition of the fungus involves Dectin-1, a surface pattern recognition receptor, and the subsequent activation of the macrophage respiratory burst function, triggering the elimination of the phagocytosed fungus (8, 9). This interplay emphasizes the multifaceted role of macrophages in orchestrating host defense mechanisms against fungal pathogens (10).

Autophagy, an intrinsic and dynamic cellular process, involves the lysosomal degradation of damaged, aged, or surplus biomolecules and organelles, releasing small molecules for cellular recycling (11). This crucial mechanism plays a pivotal role in safeguarding the body’s immune system against microbial invasions (12). In mammals, deficiencies or disorders in autophagy mechanisms may contribute to inflammation, autoimmunity, or broader immune dysregulation (11).

Autophagy, an intrinsic cellular process, is vital for degrading damaged biomolecules and organelles, contributing to immune system defense (6). Deficiencies in autophagy mechanisms can lead to inflammation, autoimmunity, and immune dysregulation (12). Our study underscores the pivotal role of autophagy in enhancing the Candida-killing capacity of macrophages.

In conclusion, our investigation delves into the impact of glucocorticoids on macrophage phagocytosis of Candida glabrata, revealing a complex interplay involving the MTOR pathway, autophagy, and the subsequent attenuation of macrophage phagocytosis. This sheds light on a potential novel mechanism underlying glucocorticoid-mediated immunosuppression and the development of invasive candidiasis. Our findings contribute to understanding the intricate relationship between glucocorticoids, macrophages, and fungal infections, offering insights for future therapeutic strategies.

Our study holds particular relevance in the context of glucocorticoids’ widespread therapeutic use for conditions such as autoimmune diseases and severe illnesses. Despite their therapeutic benefits, the observed association between glucocorticoid usage and increased susceptibility to invasive candidiasis raises critical questions about balancing therapeutic advantages with potential immunosuppressive effects.

Furthermore, understanding the intricate molecular mechanisms involved in glucocorticoid-mediated immunosuppression can guide the development of targeted therapeutic strategies. For instance, our observation that the autophagy agonist rapamycin enhances macrophage autophagy in the presence of glucocorticoids suggests a potential avenue for therapeutic intervention. Investigating the modulation of autophagy pathways may provide novel insights into mitigating the immunosuppressive effects associated with glucocorticoid therapy.

In summary, our study elucidates the impact of glucocorticoids on macrophage-mediated immune responses against Candida glabrata. The revealed inhibition of the MTOR pathway and autophagy, along with impaired phagocytosis, establishes a foundation for further exploration of the complex interplay between glucocorticoids and immune defense mechanisms. By unraveling these mechanisms, we aim to contribute valuable knowledge that may inform future therapeutic strategies to minimize the risk of invasive candidiasis in individuals undergoing glucocorticoid treatment.

## Materials and methods

### Cell line

The murine monocyte-macrophage cell line RAW264.7 (obtained from Guangzhou Ye Shan Biotechnology Co.) was utilized in this study. The RAW264.7 cell line used in this study was derived from BALB/c mice, specifically isolated from peritoneal macrophages. Cells were cultured in DMEM supplemented with penicillin-streptomycin and maintained at 37°C in a 5% CO^2^ atmosphere.

### Reagents

Fetal bovine serum (Hyclone, SH30087.01), DMEM-High Glucose Culture Medium (Hyclone, SH30022.01B), Penicillin-Streptomycin (Hyclone, SH30010), PBS Phosphate Buffered Solution (Hyclone, SH30256.01B). Sabouraud Dextrose Agar Plates (Guangdong Huan Kai Microbial Technology Co, CP0170). DHR123 (Sigma, D1054). Calcofluor White (Sigma, 18909-F). Dexamethasone (Source Leaf Biotech, S17003). Rapamycin (Cell Signaling, 9904S). Bafilomycin A1 (Selleck, S1413). Mouse IL-6 (Interleukin-6) ELISA Kit (Solarbio, SEKM-0007). Mouse TNF-α (Tumor Necrosis Factor-alpha) ELISA Kit (Solarbio, SEKM-0034). Anti-LC3B Antibody - Autophagosome Marker (Abcam, ab48394). Anti-mTOR (phospho S2448) Antibody [EPR426(2)] (Abcam, ab109268). p-mTOR Antibody (296.Ser 2481) (Santa Cruz, sc-293132). Phospho-p70 S6 Kinase (Thr389) (108D2) Rabbit mAb (Cell Signaling Technology, 9234P). Phospho-p70 S6 Kinase (Ser371) Antibody (Cell Signaling Technology, 9208T). Phospho-Raptor (Ser792) (E4V6C) Rabbit mAb (Cell Signaling Technology, 89146). Phospho-S6 Ribosomal Protein (Ser240/244) (D68F8) XP^®^ Rabbit mAb (Cell Signaling Technology, 5364). Phospho-S6 Ribosomal Protein (Ser235/236) (D57.2.2E) XP^®^ (Cell Signaling Technology, 4858). Phospho-4E-BP1 (Thr37/46) (236B4) Rabbit mAb (Cell Signaling Technology, 2855T). AO-EB Dual Staining Kit (Solarbio, CA1140). Gelatin (Sigma, G1393). Premo™ Autophagy Sensor (Thermo Fisher Scientific, P36235).

### Candida culture and infection

Candida glabrata was cultured in SDA (Sabouraud Dextrose Agar) medium and incubated at 37°C for 24 hours. Petri dishes were subsequently rinsed with sterile saline, and the liquid containing Candida glabrata was collected into centrifuge tubes. After centrifugation, the cells were ground, quantified using a haematocrit technical plate, and finally prepared into a suspension with a concentration of 10^8^ CFU (Colony-Forming Unit)/ml. The Candida glabrata suspensions were subjected to heat inactivation by incubating in a water bath at 56°C for 30 minutes. The inactivated Candida glabrata suspensions were stained with 0.1 mg/mL CFW (Calcofluor White) for 30 minutes at room temperature, washed twice with PBS, and utilized for fluorescence microscopy. For Candida glabrata spore preparation, spores were incubated with 30 µmol/L DHR123 (Dihydrorhodamine 123) at 37°C for 30 minutes, followed by centrifugation, washing with PBS, heat inactivation, and subsequent preparation for phagocytosis detection using flow cytometry.

### Fluorescence microscopy

Mouse monocyte-macrophage leukemia cells Raw264.7 (10^5^/ml) were co-cultured with Candida glabrata stained with CFW (10^6^CFU/ml) for 30 minutes, 1 hour, 2 hours, and 4 hours. At each time point, three randomly selected fields of view were captured, and the phagocytosis rate of Candida glabrata by macrophages was assessed. The percentage of cells phagocytosing more than three fungi was quantified under a 40x microscope. The phagocytosis rate was calculated as the number of macrophages phagocytosing Candida glabrata divided by the total number of macrophages, multiplied by 100%. The percentage of Candida glabrata with more than three fungi was determined as the number of macrophages phagocytosing three or more Candida glabrata divided by the total number of macrophages, multiplied by 100%.

### Flow cytometry

Mouse monocyte-macrophage leukemia cells Raw264.7 (10^5^/ml) were co-cultured with DHR123-labeled Candida glabrata (10^6^ CFU/ml) for various time points (30 min, 1 h, 2 h, and 4 h). Gating based on FSC (Forward Scatter) and SSC (Side Scatter), phagocytosis was assessed, and 10,000 cells were collected per sample. The acquired data were analyzed using FlowJo to determine the proportion of cells positive for fluorescence and the Mean Fluorescence Intensity (MFI) for statistical evaluation.

### Experimental group

Mouse monocyte macrophage leukemia cells Raw264.7 (10^5^/ml) were co-cultured with inactivated Candida glabrata (10^6^ CFU/ml) to generate the Candida suspension. Dexamethasone was introduced to the Candida suspension at a concentration of 200 ng/µl, followed by the addition of 100 µM rapamycin. To establish a baseline, Raw264.7 cells infected without Candida glabrata were designated as the control group (“C”). The experimental groups included Raw264.7 cells infected with Candida glabrata, labeled as the “Cg” group. Measurements were conducted at 6, 12, and 24 hours of co-cultivation. Another group, designated as “DEX+Cg”, consisted of Raw264.7 cells infected with Candida glabrata and pre-treated with dexamethasone, with measurements taken at the same time intervals. The third group, “DEX+Rapa+Cg”, involved Raw264.7 cells infected with Candida glabrata and pre-treated with dexamethasone and rapamycin, with measurements at 6, 12, and 24 hours of co-cultivation.

In our study, Raw264.7 cells were co-cultured with DHR123-labeled Candida glabrata at a ratio of 1:10. The untreated cell group served as the control, denoted as “1:10Cg”. Raw264.7 cells were seeded at a density of 1.5×10^5^/dish and pretreated for 24 hours under different conditions: the control group (“1:10Cg”), and experimental groups: “10DEX1d+1:10Cg”, “100DEX1d+1:10Cg”, and “200DEX1d+1:10Cg”, with dexamethasone concentrations of 10ng/µl, 100ng/µl, and 200ng/µl, respectively. Additionally, Raw264.7 cells were pretreated with 100 µmol/L rapamycin for 12 hours and then co-cultured with Candida glabrata. This group of cells was labeled as “Rapa 12h+1:10Cg”. Macrophage phagocytosis of Candida glabrata was investigated at different time points (0.5h, 1h, 2h, and 4h) during co-culture.

### ELISA

Levels of IL-6 and TNF-α in the supernatant from Raw264.7 macrophage cell cultures and Candida suspension were assessed using ELISA (Solarbio, SEKM-0034) and ELISA (Solarbio, SEKM-0007). The Candida suspension was co-treated for 6h, 12h, and 24h, respectively. Direct supernatant (100 µL) and diluted samples (20-50 times) were subjected to analysis on ELISA plates. Cytokine concentrations were determined in accordance with the manufacturer’s instructions.

### Western blot analysis

Protein extraction from the Candida suspension was performed using Radio-Immunoprecipitation Assay (RIPA) lysate. The protein concentration was determined, and the collected protein samples were supplemented with an appropriate amount of concentrated Sodium Dodecyl Sulfate Polyacrylamide Gel Electrophoresis (SDS-PAGE) Protein Sampling Buffer. The mixture was heat-treated in a boiling water bath for 3-5 minutes to achieve complete protein denaturation. After cooling to room temperature, the protein samples were directly loaded into the SDS-PAGE wells. Separated proteins from the gel were transferred onto a membrane (Polyvinylidene difluoride - PVDF) via electroblotting. Non-specific binding sites on the membrane were blocked by incubating it with a blocking solution. The membrane was then incubated with a primary antibody specific to the target protein. Following this, the membrane was washed to remove unbound primary antibodies. Subsequently, the membrane was incubated with a secondary antibody conjugated to an enzyme, and unbound secondary antibodies were washed away. Protein detection was achieved using BeyoECL Plus reagents. The resulting film was scanned or photographed and analyzed by Quantity One for determining the molecular weight and net optical density values of the target bands.

### AO/EB staining

A working solution of Acridine Orange (AO) and Ethidium Bromide (EB) was prepared by mixing the two dyes in a ratio of 1:1. After treatment, Raw264.7 cells were harvested and washed with PBS. The AO/EB working solution was then added to the cell pellet, and the cells were incubated for 2-5 minutes at room temperature in the dark. Stained cells were visualized under a fluorescence microscope using appropriate filter sets for AO (green fluorescence) and EB (red fluorescence). Digital images of the stained cells were captured using a fluorescence microscope equipped with a camera. The percentage of viable cells was quantified based on the intensity and morphology of the fluorescence signals.

### Autophagy monitoring assay

Add 2 μL of Premo™ LC3B-GFP Kit Autophagy Sensor (Component A) medium per 10,000 Raw264.7 cells and incubate the cells at 37°C for 16 hours. The different stages of autophagy were monitored using the Premo™ Autophagy Sensor GFP-LC3B Kit (Thermo, P36235) following the manufacturer’s instructions.

### Confocal microscopy

Utilizing laser confocal microscopy, we conducted a comprehensive examination of macrophage fluorescence, emphasizing both quantification and spatial distribution subsequent to transfection with GFP-LC3B baculovirus. Moreover, the visualization of macrophage viability was achieved through the observation of live and dead cells stained with Acridine Orange and Ethidium Bromide (AO/EB) using laser confocal microscopy.

### Statistical analysis

Statistical analysis was conducted using GraphPad Prism 5.0 software (GraphPad Software, San Diego, CA). ELISA, AO/EB staining, Western blot, and macrophage phagocytosis of Candida glabrata were assessed, with data presented as the mean ± standard error of the mean (SEM). One-way analysis of variance (ANOVA) was employed to compare data among three or more groups. Differences were considered statistically significant when the p-value was less than 0.05.

## Results

### Dexamethasone and rapamycin exhibit inhibitory effects on macrophage secretion of proinflammatory cytokines, while dexamethasone additionally enhances macrophage survival

**Figure 1:**
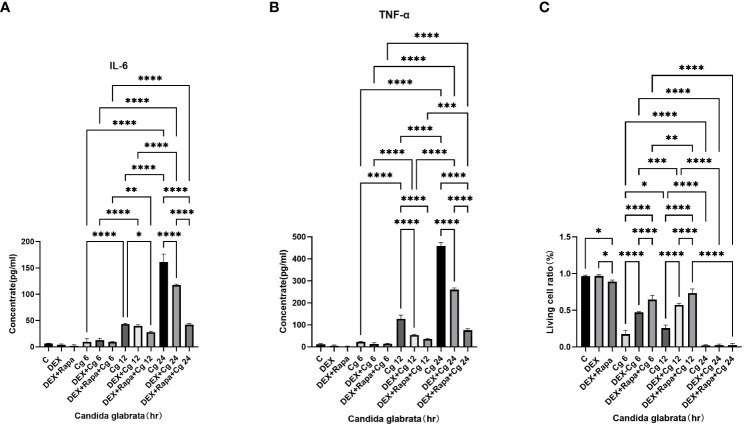
TNF-α **(A)** and IL-6 **(B)** levels in cell supernatants were assessed through ELISA. The proportion of viable cells **(C)** was determined using the AO/EB staining method. Data are shown as the mean ± SEM of three independent experiments. *p < 0.05; **p < 0.01; ***p < 0.001; ****p < 0.0001.

Under Candida glabrata stimulation, the secretion of TNF-α and IL-6 by macrophages exhibited a time-dependent increase, demonstrating statistical significance (P<0.05, **Figures 1A, B**). This indicates that Candida glabrata induces the secretion of pro-inflammatory factors TNF-α and IL-6 by macrophages. To explore the impact of glucocorticoids on intrinsic immunity during Candida glabrata phagocytosis by macrophages, cells were pre-treated with the glucocorticoid dexamethasone. The co-culture of macrophages with Candida glabrata for 24 hours revealed a significant inhibition of TNF-α and IL-6 expression levels by glucocorticoids (P<0.05, **Figures 1A, B**). This suggests that glucocorticoids suppress intrinsic immunity during Candida glabrata infection. To investigate whether glucocorticoids inhibit intrinsic immunity by influencing macrophage autophagy, macrophages were pre-treated with the autophagy inducer rapamycin. Under the influence of glucocorticoids, rapamycin further decreased the expression levels of IL-6 and TNF-α when macrophages were co-cultured with Candida glabrata for 24 hours (P<0.05, **Figures 1A, B**).

**Figure 2:**
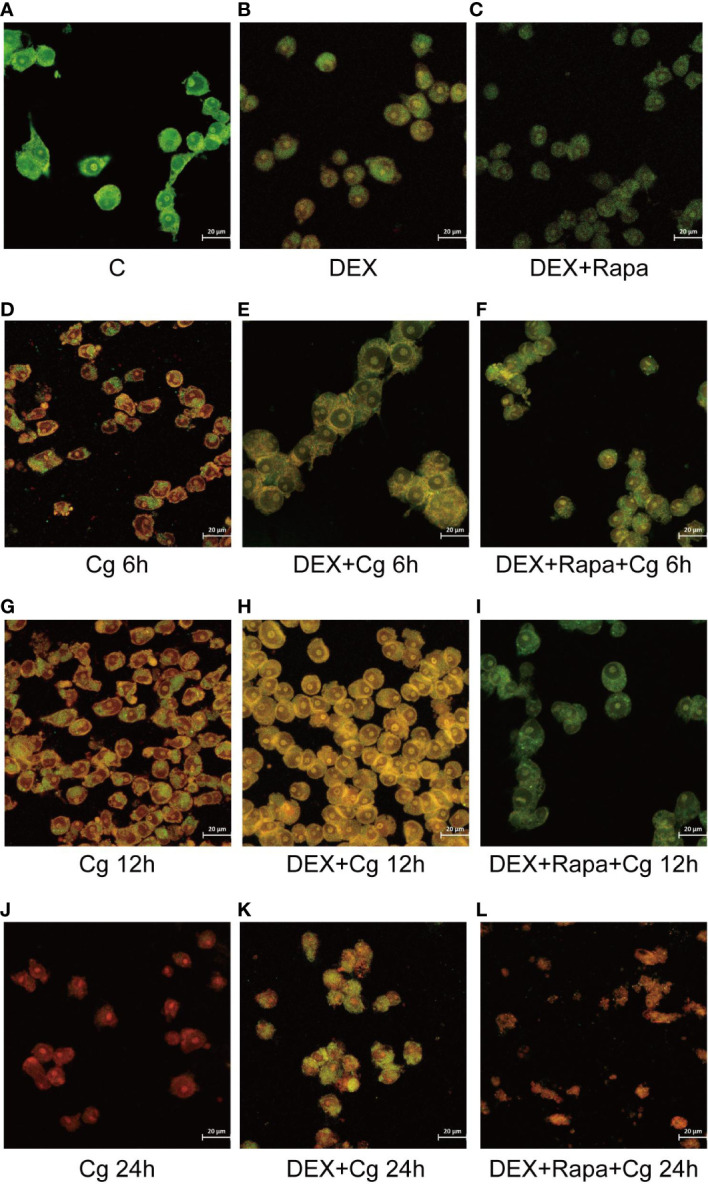
Laser confocal microscopy was employed to observe AO/EB staining in both dead and living cells. AO led to the emission of green light in chimeric DNA double strands, while others emitted red light. EB emitted red light uniformly without base-specificity. Raw264.7 cells not infected with Candida glabrata as control group **(A)**. Raw264.7 cells pre-treated with dexamethasone **(B)**. Raw264.7 cells pre-treated with dexamethasone and rapamicin **(C)**. Raw264.7 cells infected with Candida glabrata, co-cultured for 6 hours **(D)**. Raw264.7 cells pre-treated with dexamethasone and infected with Candida glabrata, co-cultured for 6 hours **(E)**. Raw264.7 cells infected with Candida glabrata and pre-treated with dexamethasone and rapamycin, co-cultured for 6 hours **(F)**. Raw264.7 cells infected with Candida glabrata, co-cultured for 12 hours **(G)**. Raw264.7 cells pre-treated with dexamethasone and infected with Candida glabrata, co-cultured for 12 hours **(H)**. Raw264.7 cells infected with Candida glabrata and pre-treated with dexamethasone and rapamycin, co-cultured for 12 hours **(I)**. Raw264.7 cells infected with Candida glabrata, co-cultured for 24 hours **(J)**. Raw264.7 cells pre-treated with dexamethasone and infected with Candida glabrata, co-cultured for 24 hours **(K)**. Raw264.7 cells infected with Candida glabrata and pre-treated with dexamethasone and rapamycin, co-cultured for 24 hours **(L)**.

The viability of macrophages significantly decreased (P<0.01, **Figures 1C**, **2**) after 24 hours of co-culture with Candida glabrata. This observation aligns with previous findings, indicating a notable surge in IL-6 and TNFα secretion by macrophages at the 24-hour co-culture mark compared to earlier time points (**Figures 1A, B**). In contrast, when macrophages were co-cultured with Candida glabrata for 6 and 12 hours, treatment with glucocorticoids resulted in a remarkable increase in macrophage viability (P<0.01, **Figures 1C**, **2**). Furthermore, under the influence of glucocorticoids, rapamycin treatment significantly enhanced the macrophage viability (P<0.01, **Figures 1C**, **2**).

Excessive production of proinflammatory factors has been associated with macrophage apoptosis. Both rapamycin and glucocorticoids are known for their robust immunosuppressive properties. Therefore, it is postulated that glucocorticoids and rapamycin function to inhibit macrophage apoptosis during the phagocytosis of Candida glabrata.

### Dexamethasone hinders the phagocytosis of Candida glabrata by macrophages

**Figure 3:**
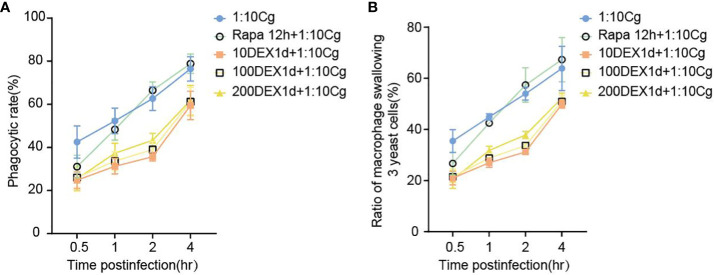
The phagocytosis rate **(A)** of Candida glabrata and the proportion of cells engulfing more than 3 yeast cells **(B)** by Raw264.7 cells were assessed at 0.5h, 1h, 2h, and 4h of co-culture, respectively. Data are shown as the mean ± SEM of three independent experiments.

**Figure 4:**
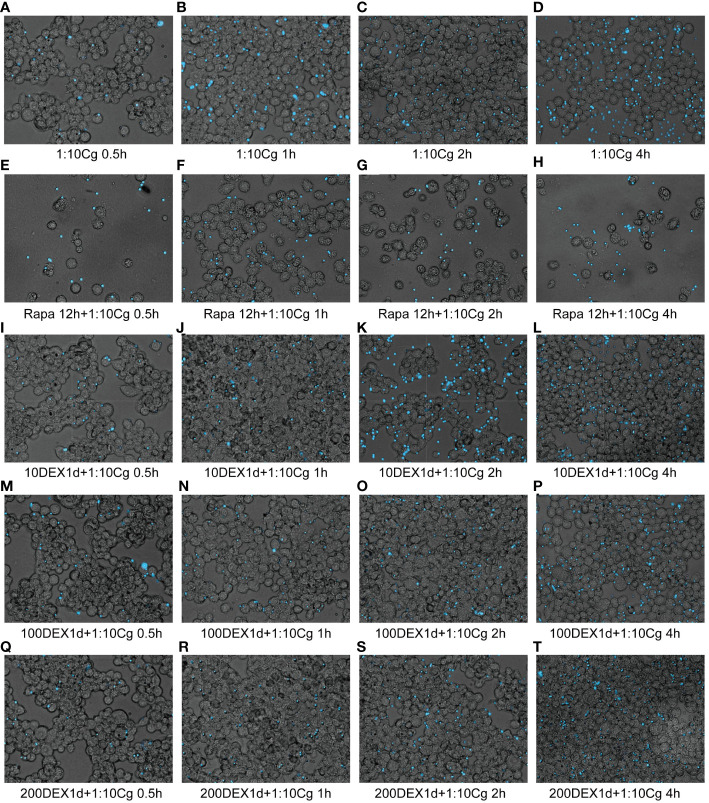
Phagocytosis of Candida glabrata by macrophages observed by fluorescence microscopy. The untreated Raw264.7 cells, co-cultured with DHR123 labeled Candida glabrata at a 1:10 ratio, were examined at 0.5h **(A)**, 1h **(B)**, 2h **(C)**, and 4h **(D)**. Raw264.7 cells pretreated with 100 µmol/L rapamycin for 12 hours and then co-cultured with Candida glabrata were studied at 0.5h **(E)**, 1h **(F)**, 2h **(G)**, and 4h **(H)**. Raw264.7 cells, pre-treated for 24 hours with dexamethasone concentrations of 10ng/µl, were assessed at 0.5h **(I)**, 1h **(J)**, 2h **(K)**, and 4h **(L)**. Raw264.7 cells pre-treated for 24 hours with dexamethasone concentrations of 100ng/µl were investigated at 0.5h **(M)**, 1h **(N)**, 2h **(O)**, and 4h **(P)**. Raw264.7 cells pre-treated for 24 hours with dexamethasone concentrations of 200ng/µl were examined at 0.5h **(Q)**, 1h **(R)**, 2h **(S)**, and 4h **(T)**.

**Figure 5:**
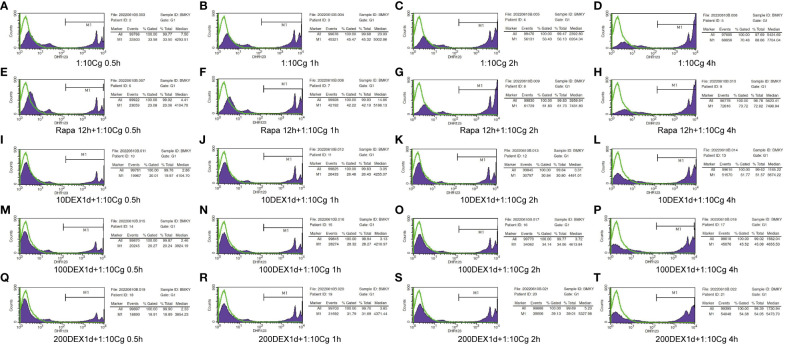
Phagocytosis of Candida glabrata by macrophages detected by flow cytometry. Add behind: The untreated Raw264.7 cells, co-cultured with DHR123 labeled Candida glabrata at a 1:10 ratio, were examined at 0.5h **(A)**, 1h **(B)**, 2h **(C)**, and 4h **(D)**. Raw264.7 cells pretreated with 100 µmol/L rapamycin for 12 hours and then co-cultured with Candida glabrata were studied at 0.5h **(E)**, 1h **(F)**, 2h **(G)**, and 4h **(H)**. Raw264.7 cells, pre-treated for 24 hours with dexamethasone concentrations of 10ng/µl, were assessed at 0.5h **(I)**, 1h **(J)**, 2h **(K)**, and 4h **(L)**. Raw264.7 cells pre-treated for 24 hours with dexamethasone concentrations of 100ng/µl were investigated at 0.5h **(M)**, 1h **(N)**, 2h **(O)**, and 4h **(P)**. Raw264.7 cells pre-treated for 24 hours with dexamethasone concentrations of 200ng/µl were examined at 0.5h **(Q)**, 1h **(R)**, 2h **(S)**, and 4h **(T)**.

In the context of glucocorticosteroid influence on macrophages, the phagocytosis rate of Candida glabrata by macrophages and the proportion of macrophages engulfing more than three yeast cells significantly increased at 4h compared to 0.5h, 1h, and 2h (P<0.05, **Figure 3B**). The number of Candida glabrata phagocytosed by macrophages demonstrated a time-dependent escalation (**Figure 3**). Dexamethasone treatment hindered the phagocytosis of Candida glabrata by macrophages (P<0.05, **Figures 3**–**5**), but the phagocytosis rate was not distinct under varying glucocorticosteroid concentrations (**Figure 3A**). The autophagy inducer rapamycin had no substantial impact on the phagocytosis of Candida glabrata by macrophages (**Figure 3**). For co-culture durations of 0.5h and 1h, rapamycin marginally inhibited the phagocytosis, with no discernible difference (**Figure 3**). However, for 2h and 4h co-culture, rapamycin enhanced the phagocytosis, yet without significant differentiation (**Figure 3**).

### Dexamethasone exerts inhibitory effects on macrophage autophagy, and this inhibition is effectively reversed by the administration of rapamycin

Dexamethasone exhibited a significant inhibitory effect on the phagocytosis of Candida glabrata by macrophages. Interestingly, the impact of the autophagy inducer rapamycin on phagocytosis displayed a dual nature, showing both inhibitory and promotional effects on Candida glabrata phagocytosis by macrophages (**Figure 3**). Consequently, further investigation is warranted to elucidate the specific interactions between dexamethasone and autophagy in this context.

**Figure 6:**
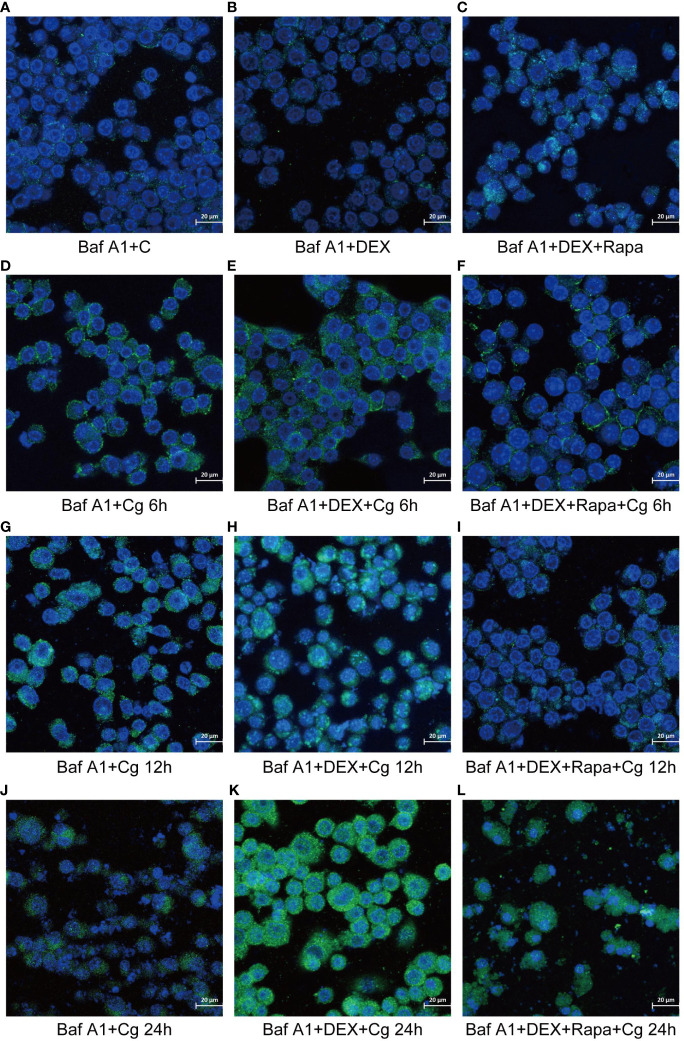
Laser confocal microscopy was employed to observe the distribution of GFP-LC3B, a fusion protein of Green Fluorescent Protein (GFP) and LC3B, in macrophages. In the images, LC3B was represented in green fluorescence, and the cell nuclei were stained in blue with DAPI. This approach allowed for a detailed examination of the subcellular localization and distribution of GFP-LC3B within the macrophages, providing valuable insights into the dynamics of autophagy in the studied cellular context. Raw264.7 cells not infected with Candida glabrata as control group **(A)**. Raw264.7 cells pre-treated with dexamethasone **(B)**. Raw264.7 cells pre-treated with dexamethasone and rapamicin **(C)**. Raw264.7 cells infected with Candida glabrata, co-cultured for 6 hours **(D)**. Raw264.7 cells pre-treated with dexamethasone and infected with Candida glabrata, co-cultured for 6 hours **(E)**. Raw264.7 cells infected with Candida glabrata and pre-treated with dexamethasone and rapamycin, co-cultured for 6 hours **(F)**. Raw264.7 cells infected with Candida glabrata, co-cultured for 12 hours **(G)**. Raw264.7 cells pre-treated with dexamethasone and infected with Candida glabrata, co-cultured for 12 hours **(H)**. Raw264.7 cells infected with Candida glabrata and pre-treated with dexamethasone and rapamycin, co-cultured for 12 hours **(I)**. Raw264.7 cells infected with Candida glabrata, co-cultured for 24 hours **(J)**. Raw264.7 cells pre-treated with dexamethasone and infected with Candida glabrata, co-cultured for 24 hours **(K)**. Raw264.7 cells infected with Candida glabrata and pre-treated with dexamethasone and rapamycin, co-cultured for 24 hours **(L)**.

**Figure 7:**
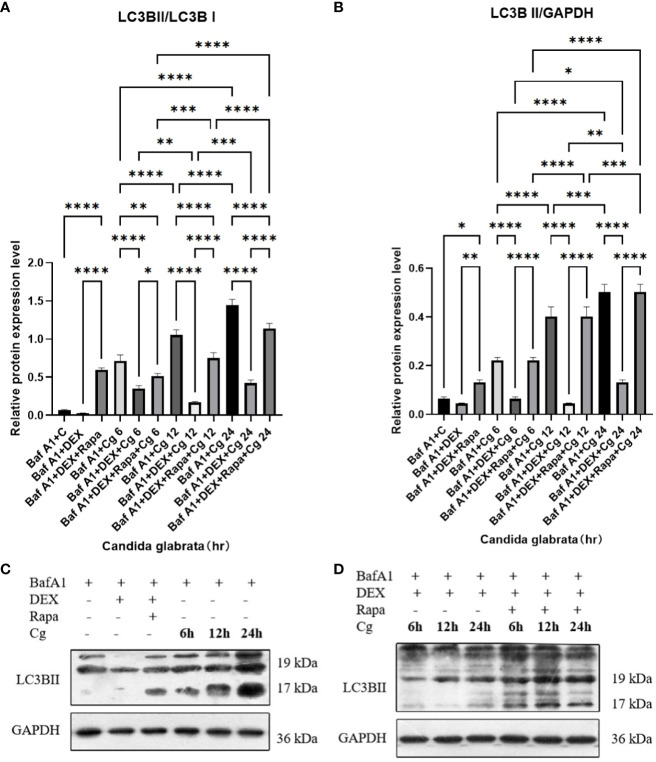
The evaluation of macrophage autophagy, represented by LC3BII/LC3BI **(A)** and LC3BII/GAPDH **(B)**, was conducted through Western blot analysis. Western blot analysis was performed to detect the expression of LC3BII protein in Raw264.7 cells for groups "C", "DEX", "DEX+Rapa", "Cg 6h", "Cg 12h" and "Cg 24h" **(C)**. Western blot analysis was conducted to assess the expression of LC3BII protein in Raw264.7 cells for groups "DEX+Cg 6h", "DEX+Cg 12h", "DEX+Cg 24h", "DEX+Rapa+Cg 6h", "DEX+Rapa+Cg 12h" and "DEX+Rapa+Cg 24h" **(D)**. Data are shown as the mean ± SEM of three independent experiments. *p < 0.05; **p < 0.01; ***p < 0.001; ****p < 0.0001.

During cellular autophagy, the transition of the LC3BI (LC3 cytoplasmic phenotype) to a LC3BII (LC3 membrane phenotype) occurs. This process, impeded by the BAF-A1 (lysosomal inhibitor bafilomycin-A1) at the final step of autophagy, leads to the LC3BII/LC3BI or LC3BII/GAPDH(Glyceraldehyde 3-phosphate dehydrogenase) ratio, serving as an indicator of autophagy levels. The ratio positively correlates with the extent of autophagy. LC3B predominantly localizes to the cell membrane of macrophages(**Figure 6A**). Under Candida glabrata stimulation, dexamethasone significantly reduced the accumulation of LC3BII in macrophages, indicating the inhibition of macrophage autophagy(P<0.01, **Figures 6**, **7**). These differences were statistically significant after co-cultivation of Candida glabrata with macrophages for 6h, 12h, and 24h (P<0.01, **Figures 6**, **7**). Additionally, the autophagy inducer rapamycin enhanced the accumulation level of LC3BII in macrophages (P<0.05, **Figures 6**, **7**), highlighting its potential to promote macrophage autophagy.

### Candida glabrata activation induces the MTOR pathway in macrophages, whereas dexamethasone treatment is observed to inhibit the MTOR pathway in macrophages

In our preceding experiments, we established that glucocorticoids hinder macrophage phagocytosis of Candida glabrata by suppressing macrophage autophagy. To delve deeper into the mechanistic link between glucocorticoids and cellular autophagy regulation through the MTOR pathway, we investigated alterations in the MTOR pathway within each macrophage group. As pivotal serine/threonine kinases, the MTOR pathway serves as a principal regulator of cellular metabolism, and its inhibition is known to induce autophagy.

**Figure 8:**
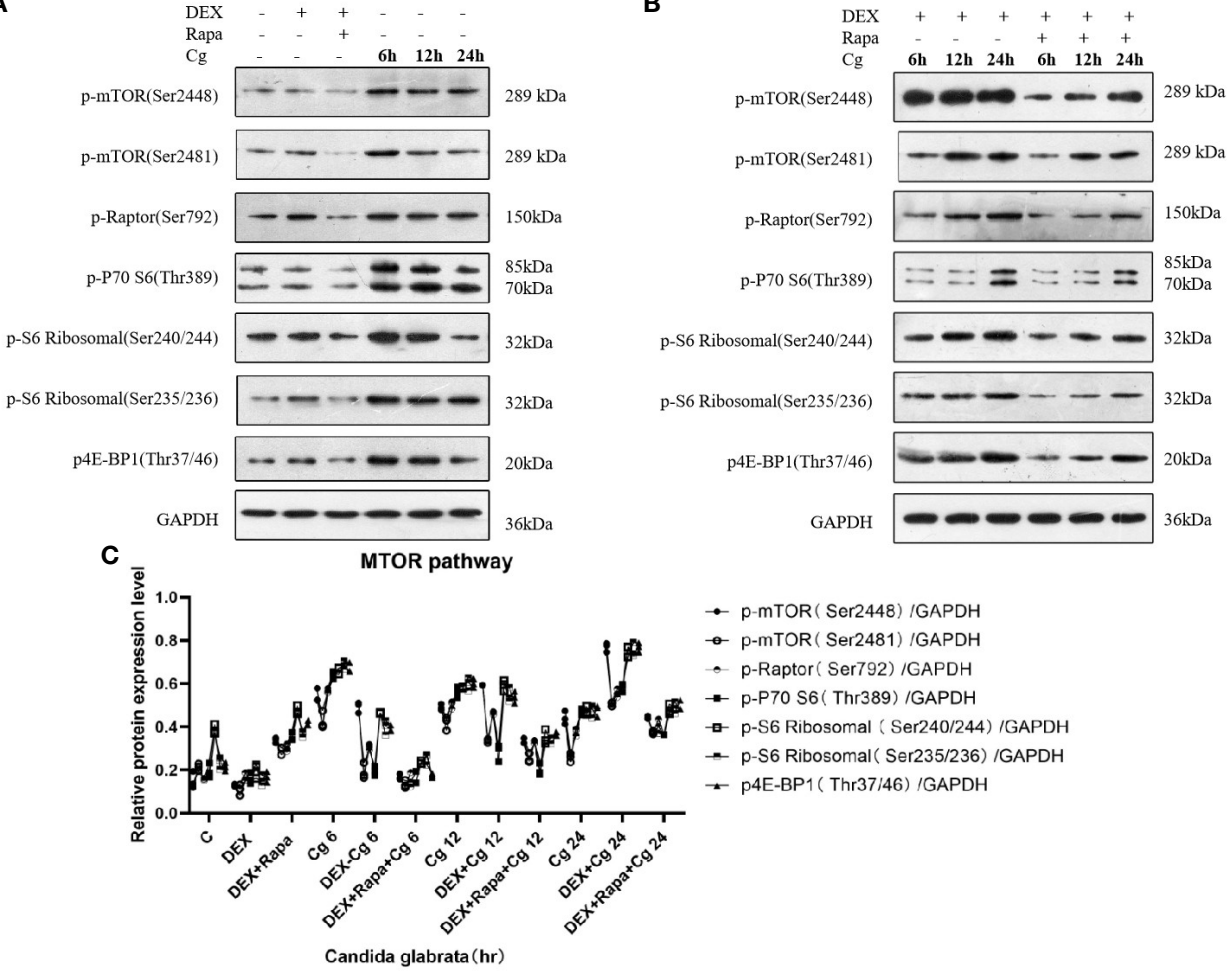
Western blot analysis was conducted to examine the expression of MTOR pathway-related proteins in Raw264.7 cells for groups "C", "DEX", "DEX+Rapa", "Cg 6h", "Cg 12h", and "Cg 24h" **(A)**. Western blot analysis was performed to assess the expression of MTOR pathway-related proteins in Raw264.7 cells for groups "DEX+Cg 6h", "DEX+Cg 12h", "DEX+Cg 24h", "DEX+Rapa+Cg 6h", "DEX+Rapa+Cg 12h", and "DEX+Rapa+Cg 24h" **(B)**. The relative protein expression levels of MTOR pathway-related proteins were compared among different cell groups **(C)**.

Upon co-culturing Candida glabrata with macrophages, we observed that Candida glabrata triggered the activation of the MTOR pathway (**Figure 8**). However, this activation diminished over time. Conversely, when Candida glabrata and macrophages were co-cultured and subjected to glucocorticoid stimulation, the inhibitory impact of glucocorticoids on the MTOR pathway showed a time-dependent reduction, and by 24 hours, glucocorticoids exhibited an activating effect on the MTOR pathway (**Figure 8**). Furthermore, under glucocorticoid stimulation, co-culturing Candida glabrata with macrophages and applying rapamycin resulted in the inhibition of the MTOR pathway (**Figure 8**).

## Discussion

Glucocorticoids find extensive use in the treatment of immune disorders and endocrine system diseases. However, it is noteworthy that glucocorticoid therapy presents a substantial risk factor for candidaemia (13). Glucocorticoids have been observed to stimulate the proliferation of Candida in the cornea, concurrently diminishing neutrophil infiltration. Moreover, glucocorticoids impede the formation of neutrophil extracellular traps, culminating in an exacerbation of fungal keratitis (3).

Candida glabrata, a conditionally pathogenic organism prevalent in mucous membranes, poses a significant risk for systemic fungal infections, particularly in immunocompromised individuals, those undergoing glucocorticoid therapy, or utilizing catheters (14). Notably, systemic fungal infections attributable to Candida glabrata represent the second most frequent among Candida genera in both the United States and Europe (15). In contrast to Candida albicans, Candida glabrata exhibits heightened resistance to the antifungal effects of reactive oxygen species (ROS) secreted by macrophages (16). This increased resistance may contribute to the elevated mortality observed in patients with Candida glabrata candidemia (17).

In the immune defense against fungal infections, macrophages play a pivotal role as primary effectors in eliminating Candida. Recognition of fungal-pathogen-associated molecular patterns (PAMPs) by pattern recognition receptors (PRRs) on the macrophage cell membrane initiates the phagocytic process. Subsequently, nascent phagosomes undergo fusion with lysosomes, maturing into phagolysosomes within macrophages. These phagolysosomes create a hostile environment lethal to Candida, contributing to fungal clearance. Moreover, macrophages engage in LC3-associated phagocytosis (LAP), a mechanism that aids in combating fungi (18). Additionally, monocytes and macrophages play a crucial role in recruiting neutrophils, further enhancing the clearance of Candida. This collaborative immune response underscores the complexity and effectiveness of the host defense against fungal pathogens. Understanding these intricate interactions provides insights into potential therapeutic strategies for managing fungal infections in clinical settings (19). Despite the phagocytosis by macrophages, Candida glabrata exhibits resilience and the ability to survive and replicate within these immune cells. This survival is attributed to various mechanisms, including the modulation of phagolysosomes, regulation of phagolysosomal pH, adaptation to the antimicrobial activities of macrophages, and the ability to adjust to the challenging nutrient environment within macrophages through autophagy (20, 21). The intricate strategies employed by Candida glabrata to evade the antimicrobial defenses of macrophages highlight the sophistication of the fungal pathogen’s immune evasion mechanisms. Further elucidating these mechanisms is crucial for developing targeted therapeutic interventions to enhance the effectiveness of macrophage-mediated clearance of Candida glabrata (20). The survival of Candida glabrata within macrophages is a key evasion strategy against immune clearance. This highlights the pathogen’s adaptability and resilience. Unraveling this interaction is vital for developing targeted therapies to counteract these evasion tactics and boost host defenses (20). Administering the MTOR pathway inhibitor rapamycin to mice with candidaemia demonstrates a noteworthy reduction in macrophage apoptosis, leading to improved overall survival (22). This observation underscores the direct protective impact of rapamycin on macrophages, suggesting its potential as a therapeutic intervention in candidaemia management (22).

Following the phagocytosis of Candida glabrata, the activation of the MTOR pathway in macrophages suggests a potential mechanism through which Candida glabrata may inhibit cellular autophagy. It is noteworthy that autophagy serves as a crucial self-protection mechanism in organisms and plays a pivotal immunomodulatory role in countering environmental stressors, particularly in chronic inflammation and infection models. Further investigations are imperative to elucidate the intricate interplay between Candida glabrata and the autophagic response, shedding light on potential therapeutic avenues for manipulating this pathway in immune regulation (23). The suppression of macrophage autophagy by Candida glabrata may serve as a mechanism enabling the continuous survival and replication of Candida glabrata within macrophages. Post-phagocytosis, there was a notable elevation in the secretion of IL-6 and TNFα by macrophages (P<0.05), accompanied by a significant reduction in the ratio of viable cells (P<0.05). These findings suggest that Candida glabrata stimulates the secretion of inflammatory factors by macrophages, contributing to macrophage death.

The inhibition of the Src/Syk pathway and the consequent reduction in reactive oxygen species production in macrophages, induced by glucocorticoids, disrupt the recruitment of LC3BII to the phagosomes of phagocytes that have internalized Aspergillus spp. This disruption, specifically the inhibition of LC3-associated phagocytosis (LAP), could be a contributing factor to the pathogenesis of invasive Aspergillus under glucocorticoid induction (24). Consistent with these findings, glucocorticoids were observed to inhibit LAP in macrophages engulfing Candida glabrata (P<0.05). Intriguingly, glucocorticoids inhibited the MTOR pathway in macrophages that had engulfed Candida glabrata, and this inhibition decreased with time, displaying activation of the MTOR pathway at 24h. Rapamycin partially mitigated the inhibition of LAP by glucocorticoids in macrophages engulfing Candida glabrata. The intricate interplay of various intracellular signaling pathways, including MTOR, AMPK (AMP-activated Protein Kinase), and JNK (c-Jun N-terminal Kinase), in autophagy suggests that glucocorticoids may exert their effects on macrophage autophagy through multiple pathways beyond MTOR. Further investigation is warranted to unravel the comprehensive regulatory network governing macrophage autophagy in response to glucocorticoids.

In the current investigation, glucocorticoids were identified to impede the phagocytosis of Candida glabrata by macrophages. Conversely, rapamycin exhibited the potential to induce phagocytosis of Candida glabrata by macrophages over time. When macrophages engulfed Candida glabrata, both glucocorticoids and rapamycin demonstrated inhibitory effects on the secretion of pro-inflammatory factors, namely IL-6 and TNFα, subsequently leading to an increased macrophage viable cell ratio. Notably, glucocorticoids suppressed macrophage autophagy, whereas rapamycin induced autophagy in macrophages. In murine experiments, glucocorticoids were observed to induce and worsen candidaemia, while rapamycin contributed to the improved survival of mice afflicted with candidaemia (22). Hence, macrophage autophagy emerges as a crucial determinant for the effective elimination of Candida glabrata subsequent to its phagocytosis. In summary, glucocorticoids impede macrophage autophagy by inhibiting the phagocytosis of Candida glabrata. This inhibition may potentially contribute to the dissemination of Candida glabrata *in vivo*, thereby fostering the development of systemic Candida infections.

The attenuation of macrophage autophagy by glucocorticoids appears to intensify with prolonged exposure, underscoring the importance of early intervention in patients with candidaemia undergoing long-term glucocorticoid treatment—a high-risk factor. Furthermore, the potential therapeutic application of rapamycin in the treatment of candidaemia emerges as a promising avenue worth exploring.

## Data availability statement

The datasets presented in this study can be found in online repositories. The names of the repository/repositories and accession number(s) can be found in the article/supplementary material.

## Ethics statement

Ethical approval was not required for the studies on animals in accordance with the local legislation and institutional requirements because only commercially available established cell lines were used.

## Author contributions

ZY: Conceptualization, Data curation, Investigation, Software, Writing – review & editing. XW: Methodology, Supervision, Writing – original draft, Writing – review & editing. TD: Funding acquisition, Validation, Writing – review & editing. W-JZ: Formal analysis, Writing – original draft, Supervision, Writing – review & editing. HL: Formal analysis, Funding acquisition, Project administration, Resources, Validation, Visualization, Writing – original draft.
